# Polygenic risk scores for cervical HPV infection, neoplasia and cancer show potential for personalised screening: comparison of two methods

**DOI:** 10.1186/s13027-023-00561-4

**Published:** 2023-12-07

**Authors:** Anna Tisler, Anneli Uusküla, Sven Erik Ojavee, Kristi Läll, Estonian Biobank research team, Andres Metspalu, Lili Milani, Tõnu Esko, Reedik Mägi, Mari Nelis, Georgi Hudjashov, Triin Laisk

**Affiliations:** 1 Institute of Family Medicine and Public Health, University of Tartu, Tartu, Estonia; 2 Department of Computational Biology, University of Lausanne, Lausanne, Switzerland; 3 Estonian Genome Centre, Institute of Genomics, University of Tartu, Tartu, Estonia

**Keywords:** Cervical cancer, Polygenic risk score, HPV, Screening, Cervical intraepithelial neoplasia

## Abstract

The era of precision medicine requires the achievement of accurate risk assessment. Polygenic risk scores (PRSs) have strong potential for increasing the benefits of nationwide cancer screening programs. The current pool of evidence on the role of a PRS as a risk stratification model in actual practice and implementation is limited. To better understand the impact of possible method-induced variance, we constructed and validated two PRSs for cervical cancer (CC) using the Estonian Biobank female population (691 CC cases and 13,820 controls) and evaluated their utility in predicting incident cervical neoplasia (CIN), cancer, and human papillomavirus (HPV) infection using two methods (LDPred and BayesRR-RC). This study demonstrated that two genetic risk scores were significantly associated with CIN, CC, and HPV infection incidence. Independent of the method, we demonstrated that women with elevated PRS values reached the observed cumulative risk levels of CIN or CC much earlier. Our results indicated that the PRS-based discrimination rules could differ substantially when the PRSs contain similar predictive information. In summary, our analysis indicated that PRSs represent a personalized genetic component that could be an additional tool for cervical cancer risk stratification, and earlier detection of abnormalities provides invaluable information for those at high risk.

**Supplementary Information:**

The online version contains supplementary material available at 10.1186/s13027-023-00561-4.

## Introduction

Cervical cancer is the fourth most frequently diagnosed cancer and the fourth leading cause of cancer death among women, with an estimated 604,000 new cases and 342,000 deaths worldwide in 2020 [1]. Cervical cancer cumulative risk among women up to age 70 in Eastern Europe is 1.4%, which is higher than that in other high human development index (HDI) countries (1.3%) and more than twice as high as that in Western Europe (0.67%) [2]. Persistent infection with high-risk HPV (hrHPV) is proven to be a causal and necessary factor for cervical cancer development and its preceding lesion. It has been estimated that the average lifetime probability of HPV among those with at least one opposite-sex partner is 84.6%, but the risk of HPV infection progressing to cervical cancer varies according to HPV genotype, preventive behaviour and health risk factors [3, 4]. Women’s behavioural and sexual characteristics associated with a higher risk of HPV infection acquisition, persistence, and progression to precancerous and more advanced cancerous stages are well described [5]. The main risk factors reported are age at first intercourse, hormonal contraception use, number of sexual partners, parity, and smoking. Women with compromised immune systems are susceptible to persistent high-risk HPV infections, consequently encountering a significantly heightened risk of cervical abnormalities [6]. However, it is acknowledged that both various exposures and heritable factors contribute to cervical cancer development [7]. Twin and family studies have estimated the heritability of cervical cancer to be 22–64%, while the common variant heritability (proportion of phenotypic variance explained by common variants) is estimated to be as high as 36% [8]. The genetic component in the HPV-lesion-cervical cancer relationship is understudied and is supported by a modest number of studies.

Genome-wide association studies (GWASs) have become a valuable tool to describe the genetic basis for common human diseases, and in line with this, they have also identified susceptibility loci for cervical cancer [9]. Polygenic risk scores (PRSs) combine the effects of several genetic variants into one variable that can be used to assess the genetic risk of a disease for an individual. Therefore, PRSs allow grouping participants into different risk categories for disease and are also used as a covariate in epidemiological analyses. There are a number of methods for PRS calculation, and the methods differ in terms of two key criteria: which genetic variants to include and what weights to allocate to them. Often, when new PRS methods are introduced, comparisons are made between a limited set of methods, together with application to some real data examples, since there is a need to explore and quantify the variability of PRS values derived using different estimation methods on the same target sample [10].

Although recent GWASs have begun to clarify the genetic background of cervical cancer and preceding HPV infection, further studies explaining genetic susceptibility for prevalent HPV infection and whether there is an overlap in genetic factors for HPV infection and progression of cervical disease (CIN, CC) are needed. Here, to understand the impact of method-induced variance on genomic prediction of cervical cancer and HPV status, we compared two methods (LDPred and BayesRR-RC) for cervical cancer PRSs.

## Results

We identified 885 CC cases (overall mean age at recruitment 51.7 years, SD 13.4), 4,406 CIN cases (mean age at recruitment 38.4 years, SD 10.7), and 83 065 controls (mean age at recruitment 42.6 years, SD 14.2). We first used the prevalent CC cases (n = 691) and controls (n = 13,820) to select the best-performing PRSs for subsequent analyses, and these individuals were removed from further analyses.

### Selecting the best-performing PRSs

We evaluated a total of 12 PRSs calculated with two separate methods to select the best-performing PRS for each method. According to our analyses (Additional file 2: Table S1), in LDpred, the best score was for LDpred_p3.0000e.03 (OR 1.44, 95% CI 1.33–1.56), which included 2 894 555 variants (causal fraction 0.3%). In BayesRR-RC showed the strongest association (OR 1.44, 95% CI 1.33–1.57). In further analyses, we shall refer to these two PRSs as LDpred and BayesRR-RC, respectively.

**Fig. 1:**
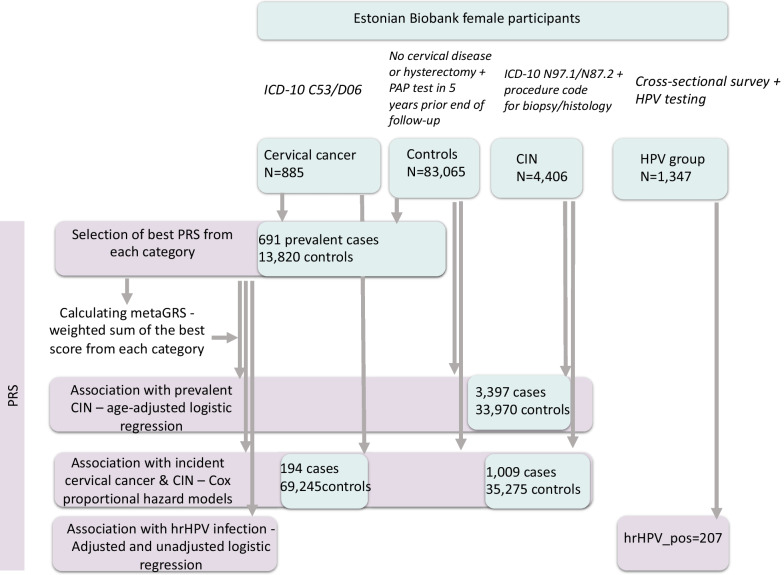
Flowchart of the study design and analysed groups

In the following analyses, the remaining cases/controls were divided as follows: incident cancer 194 cases and 69 245 controls with a mean age of 45.7 (SD 13.6) and 42.6 (SD 14.3) years, respectively; CIN 1009 cases and 35 275 controls with a mean age of 31.7 (SD 9.8) and 42.6 (SD 14.2) years, respectively; and prevalent CIN 3397 cases and 33 970 controls with a mean age of 40.0 (SD 10.0) and 42.6 (SD 14.2) years, respectively. Data on 1,347 women for association analysis with HPV infection were used (Fig. 1).

### PRS association with CIN

We found that both risk scores were significantly associated with prevalent CIN status in the case‒control subset of the EstBB cohort.

As found in the previous step, LDpred and BayesRR-RC performed relatively equally in association with prevalent CC status. The same applied with respect to prevalent CIN with an OR = 1.32 per SD, 95% CI 1.27–1.38, *p* = 1.1 × 10^–44^ with LDpred and 1.32 (95% CI 1.27–1.37), *p* = 1.3 × 10^–42^ with BayesRR-RC.

### PRS association with incident CC/CIN

Next, we evaluated the performance of the PRSs for incident CC or CIN in EstBB. Both PRSs were associated with both conditions (*p* < 0.05). For CC, the risk increased 1.32-fold per 1-SD increase in the LDpred PRS (Harrell’s C-statistic of 0.581, SE 0.020). BayesRR-RC showed a slightly lower HR of 1.25 (Harrell’s C-statistic of 0.566, SE 0.022). On the other hand, BayesRR-RC had a slightly higher HR for CIN of 1.37 (Harrell’s C-statistic 0.59, SE 0.009) compared to LDpred with an HR of 1.34 (Harrell’s C-statistic 0.582, SE 0.009).

Women in the highest 20% of genetic risk were estimated to have a 2.32 (BayesRR-RC) to 2.50 (LDpred) times greater risk of developing CC than women in the lowest 20% (Table 1). The effect was less pronounced when comparing the top 20% of women with the women below the median, resulting in a 1.58 (BayesRR-RC) to 1.66 (LDpred) times greater risk for the top 20% of women. A similar effect was observed when comparing the top 20% of women with the rest, giving hazard ratios from 1.49 (LDpred) to 1.60 (BayesRR-RC) (Table 1). Similar to CC, a clear risk gradient was observed within the risk categories for CIN. Women in the top 20% of genetic risk had an HR of 2.42 (BayesRR-RC) to 2.38 (LDpred) for incident CIN compared to women in the bottom 20%, HR of 1.91 (LDpred) to 1.98 (BayesRR-RC) compared to women below the median and HR of 1.62 (LDpred) to 1.68 (BayesRR-RC) compared to the rest of the cohort (Table 1).

**Table 1:** Hazard ratios of incident cervical cancer and cervical intraepithelial neoplasia for the two evaluated genetic risk scores

PRS	Reference group	Cervical cancer (CC)		Cervical intraepithelial neoplasia (CIN)	
		LDpred HR (95% CI)	BayesRR-RC HR (95% CI)	LDpred HR (95% CI)	BayesRR-RC HR (95% CI)
Top 40%	Remaining 60%	1.59 (1.20–2.11)	1.42 (1.07–1.88)	1.61 (1.43–1.82)	1.72 (1.51–1.95)
Top 20%	Remaining 80%	1.49 (1.08–2.04)	1.60 (1.17–2.19)	1.62 (1.42–1.86)	1.68 (1.46–1.93)
Top 20%	Bottom 20%	2.50 (1.51–4.14)	2.32 (1.43–3.74)	2.38 (1.93–2.95)	2.42 (1.95–3.00)
Top 20%	Below the median	1.66 (1.17–2.35)	1.58 (1.13–2.21)	1.91 (1.64–2.22)	1.98 (1.70–2.32)
Top 10%	Remaining 90%	1.40 (0.93–2.11)	1.62 (1.10–2.39)	1.56 (1.31–1.86)	1.71 (1.44–2.03)
Top 5%	Remaining 95%	1.63 (0.96–2.76)	1.69 (1.01–2.82)	1.68 (1.34–2.11)	2.00 (1.61–2.48)

**Fig. 2:**
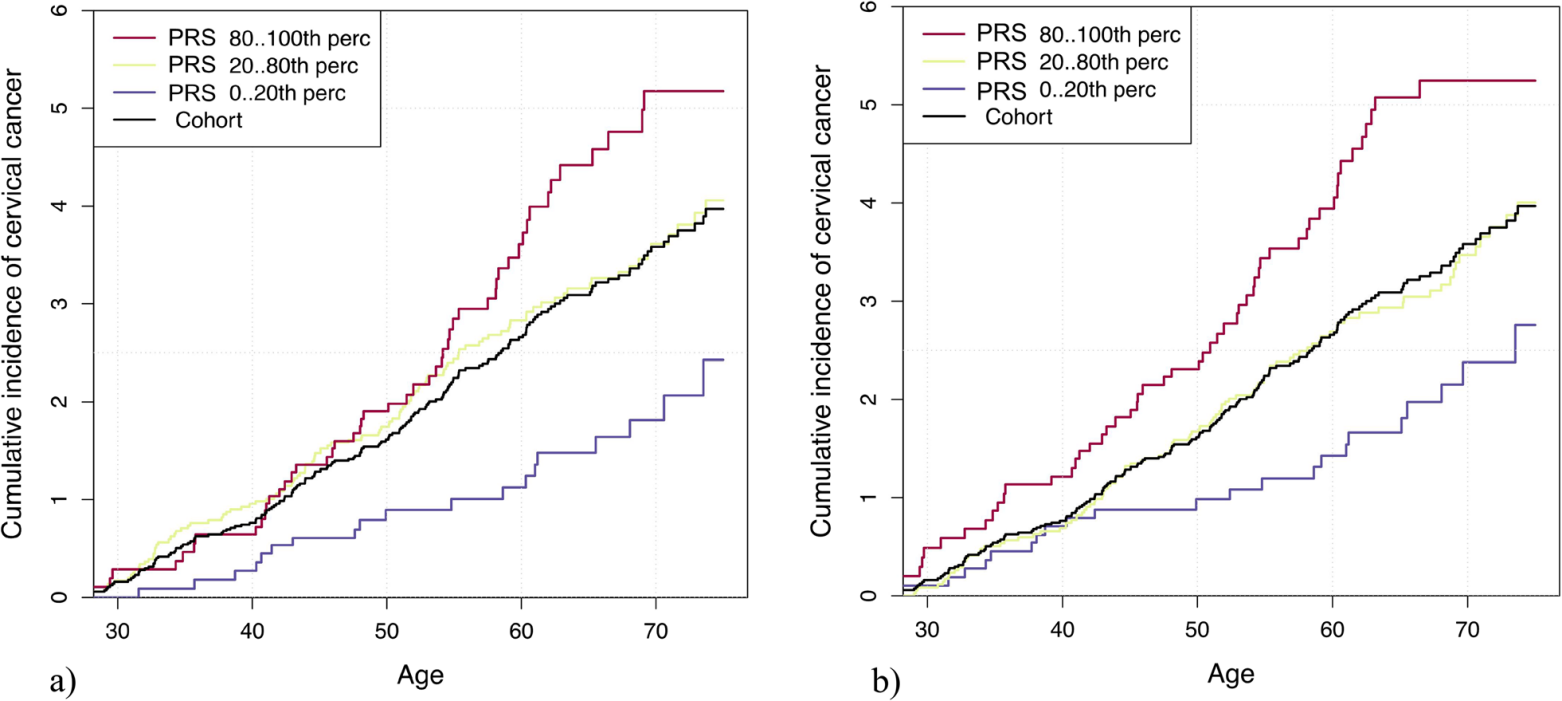
Cumulative incidence of cervical cancer (accounting for competing risks) in **a** LDpred and **b** BayesRR-RC risk categories among women aged 30–75 years

As seen in Fig. 2, the cumulative incidence of CC by age 70 was estimated to be 5.3% (95% CI 3.7–6.8) for women in the top 20% of genetic risk (as defined using LDpred), 3.7% (95% CI 2.9–4.3) for those between the 20-80th percentiles and 1.8% (95% CI 0.9–1.8) for those in the lowest 20%. The cumulative incidence in risk categories defined using BayesRR-RC was similar (5.3%, 3.5%, and 2.4%, respectively) (Fig. 2b).

**Fig. 3:**
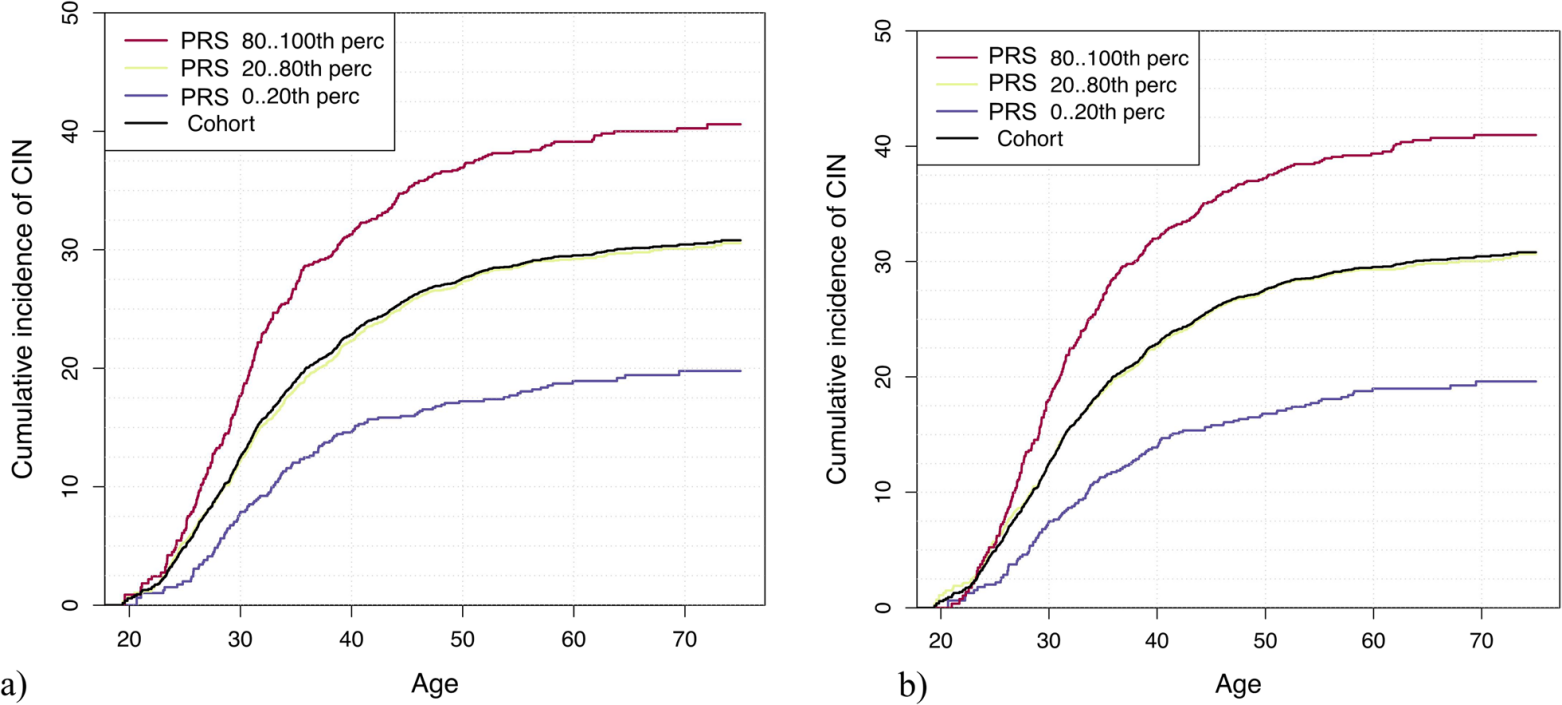
Cumulative incidence of cervical intraepithelial neoplasia (accounting for competing risks) in **a** LDpred and **b** BayesRR-RC risk categories among women aged 20–75 years

As seen in Fig. 3a, the cumulative incidence of CIN by age 50 was estimated to be 37.1% (95% CI 33.3–40.7) for women in the top 20% of genetic risk, while it was 17.2% (95% CI 14.0–20.3) among women in the bottom 20% with LDpred. The results of BayesRR-RC (Fig. 3b) were similar, with a cumulative incidence of 37.4% (95% CI 33.6–40.9) for the top 20%.

### Correlation of PRSs

**Fig. 4:**
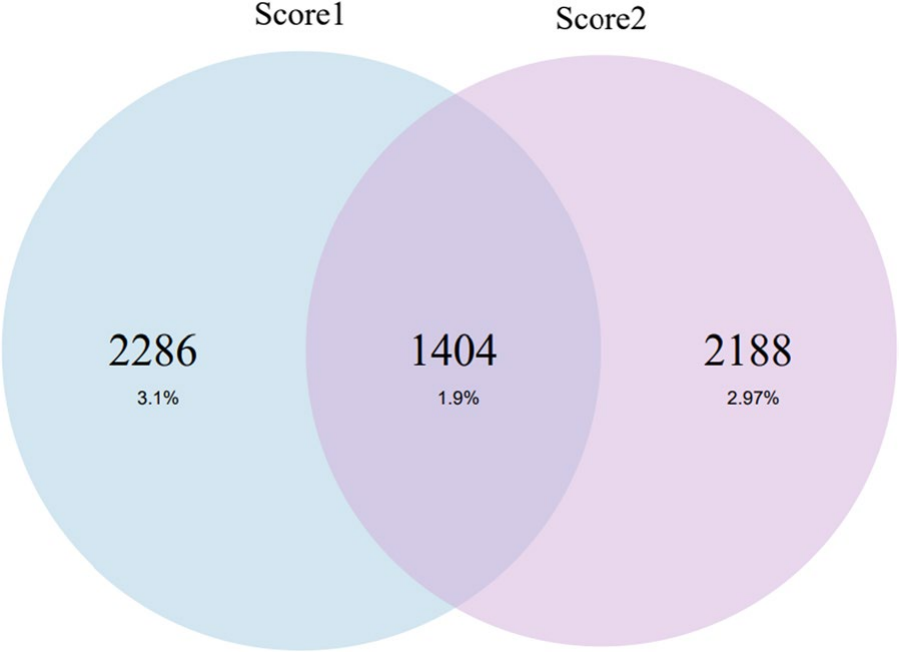
The overlap among highest-risk women (top 5%) in the Estonian Biobank according to two genetic risk scores for cervical cancer. The graph shows women who were classified as being in the top 5% with at least one of the genetic risk scores (LDpred and BayesRR-RC)

The Pearson correlation between LDpred and BayesRR-RC was 0.76. We then divided all women into two categories (high: PRS in the top 5%, not high: everyone else) based on the two PRSs. Eight percent of women belonged to the high category with at least one PRS, while 1.9% were in the top 5% with both compared PRSs (Fig. 4). Even though the scores were strongly correlated, we observed that the individual classification into the top 5% risk score category depended on a selected score and often did not overlap for a single individual. We also combined LDpred and BayesRR-RC into a further score called metaPRS (see Methods). When analysing the metaPRS in association with incident CC and CIN using the Cox proportional hazards model, the results mirrored those from the analysis of individual scores (HR 1.31 (SE 0.07), C-statistic 0.578 (SE 0.021); thus, additional results are not shown.

### Associations of risk scores with predictors of high-risk HPV infection

Both PRSs were significantly associated with high-risk HPV (hrHPV) infection, giving an adjusted OR of 1.25 (95%CI 1.08–1.44) and 1.26 (95% CI 1.09–1.47) for BayesRR-RC and LDpred respectively (Additional file 2: Table S2). We further quantified the effect of nongenetic HPV risk factors while adjusting for the PRS value, hence enabling hrHPV risk estimation conditional on genetic factors. Several nongenetic risk factors were associated with hrHPV infection (BayesRR-RC and LDpred, respectively): being single OR 1.77 (95%CI 1.29- 2.43) and 1.81 (95%CI 1.32–2.5), having secondary education rather than tertiary education OR 1.37 (95% CI 1.00–1.86) and OR 1.38 (95% CI 1.01–1.88), long term hormonal contraceptive use OR 1.63 (95%CI 1.06–2.49) and OR 1.63 (95%CI 1.01- 2.63) and number of lifetime sexual partners OR 1.04 (95%CI 1.02–1.06). The AUC for the logistic regression model that included 8 predictor variables for HPV infection was 0.682. The AUC with 8 variables and cervical cancer PRS to predict HPV status in a logistic regression model was 0.700 and 0.694 with LDpred and BayesRR-RC risk scores, respectively.

## Discussion

In our study, we demonstrated that two genetic risk scores calculated using different approaches were significantly associated with CC and CIN status in the case‒control subset of our cohort. While on average, approximately 1% of women in our dataset were diagnosed with CC by the age of 70, women in the highest five percentiles of our tested PRSs reached the same cumulative risk level by age 55, 15 years earlier. Similarly, on average, approximately 30% of women in our dataset were diagnosed with CIN by the age of 70, but women in the top 20% of genetic risk reached the same cumulative incidence before their 40^th^ birthday. Our results suggest that genetic risk estimation could be an additional tool for CC risk stratification in clinical practice, either for targeted screening or prevention practices. However, there are certain aspects that need to be considered and that are discussed in more detail below. In addition to our main findings, both tested genetic risk scores were also strong predictors of hrHPV infection, comparable to known risk factors such as marital status and the number of partners. Since the genetic risk score summarizes all the genetic risk factors for CC, we speculate that the same genetic factors associated with CC susceptibility are also associated with hrHPV infection, providing insight into HPV genetic susceptibility, which has thus far remained poorly characterized. We have shown that including PRS as a covariate induced a nominal increase in the predictive performance in addition to the 8 known risk factors. The difference was small and most of the partitions did not consider this difference statistically significant, although we did find enrichment in significance compared to the expectation.

Our results are in line with the findings by Koel et al. [11], who showed that a large part of the predictive power of PRSs for CC comes from the HLA fraction. HLA-related signalling, on the other hand, plays a central role in the course of HPV infection and may determine whether the infection is successfully cleared or persists and develops into a malignant lesion. It is possible that the PRSs capture different biological pathways or mechanisms, which is also supported by our results, in which the two scores showed very similar results in the analysis of prevalent cases but different results in the analysis of incident cases. Hence, we encourage drawing comparisons separately for prevalent and incident cases, as this could pinpoint different aspects of genetic risk prediction. In addition, we found nominal differences implying that BayesRR-RC might better reflect the CIN risk and LDpred better reflect the CC risk, it should be noted that the differences were very small, and the clinical significance of those differences is outside the scope of this study.

The correlation between the two PRSs was substantial (Pearson correlation of 0.76), which is expected given the overlap in the datasets used to estimate genome-wide effects for SNPs that were then later used to develop the scores (namely, UK Biobank data). The main difference in terms of methods is that LDpred was constructed using multiple datasets combining many marginal SNP effect estimates (one SNP at a time), whereas BayesRR-RC was obtained using a single dataset, but estimates were retrieved jointly (all SNP effects were estimated in one model). LDpred could better leverage the heterogeneity in samples, and BayesRR-RC could better leverage the genetic architecture (for example, LD structure) implications on genetic prediction, provided the training set (UK Biobank) and test set (Estonian Biobank) have relatively similar genetic architecture profiles. Nevertheless, the similarity of these two scores was further confirmed by the fact that the metaPRS combining LDpred and BayesRR-RC did not yield noticeably improved predictions compared to the scores separately. Despite the large correlation, we observed that the two scores classified high-risk individuals differently. For example, 1.9% of women in our dataset were in the top 5% with both compared PRSs, indicating that approximately 60% of the individuals in the top 5% of LDpred would not be classified as individuals in the top 5% of risk with BayesRR-RC. This demonstrates that PRS-based risk stratification could result in substantial differences across methods when identifying high-risk patients. Due to these differences, it has been suggested to provide a probability of belonging to an increased risk category instead of strict categories to account for the variability of scores [12]. More research with larger and fully characterized samples is needed to assess the utility of combining a PRS with hrHPV status and other clinical risk factors into a complex risk prediction tool.

A major strength of our study is the fact that due to the nature of data at the EstBB, we were able to include only women with a known CIN status, as we could use procedure codes in combination with disease codes to select only those women as controls who had a Pap test during the 5 years prior to this study and did not have diagnosis codes for CC or CIN. Although this approach reduced the heterogeneity of the data, it also biased the prevalence and incidence rates of the evaluated diagnoses, which means that these cannot directly be extrapolated to the general population. It is important to acknowledge that certain risk factors commonly associated with HPV, such as smoking, were not included in our analysis or measured. In addition, the analysis concerning the association between the polygenic risk score (PRS) and high-risk human papillomavirus (hrHPV) infection was limited by female participants within specific age groups (30–33, 57–60, 67–70). Age group-specific estimates directions (Additional file 2: Table S2) reflect the U-shaped hrHPV prevalence in different age groups. We observe comparable associations in terms of strength (though with overlapping confidence intervals). It is essential to interpret these findings with caution, considering the inclusion of specific age groups and the limitation of low sample numbers). In risk prediction for cancers, there is a growing interest in utilizing PRSs both in clinical practice and screening. The need for risk-stratified cervical cancer screening is evident due to the shortcomings of existing screening practices, and the promise of a better balance of benefits (preventing cancer deaths) and harms (unnecessary screenings, false-positive tests, and overdiagnosis). In case of the cervical cancer, the evidence of genetic factors involved in interactions between the host and HPV is still limited and largely unknown (except for specific variations within the human leukocyte antigen (HLA) locus on chromosome 6p21.3). Further research is needed incl. studies to identify PRS associated with cervical cancer mortality and the utility of integrating PRSs into screening practice currently is unclear [13, 14].

## Materials and methods

Data from the Estonian Biobank (EstBB) were used to compare the performance of previously published cervical cancer PRSs calculated using different approaches and to evaluate their utility in association with CC, CIN and hrHPV.

### Data source (target population) and genotyping

The Estonian Biobank (EstBB) is a population-based biobank with genotype data and health information for over 200,000 participants recruited between 2002 and 2020 [15] in its latest data freeze, which represents approximately 20% of the Estonian adult population. EstBB women were followed up from the date of EstBB entry until 31.12.2019, which is the date of the last link with the main dataset during the study period. All EstBB participants were genotyped using Illumina Global Screening Array v1.0 and v2.0 at the Genotyping Core Lab of the Institute of Genomics, University of Tartu. After genotyping, PLINK format files were created using Illumina GenomeStudio v2.0.4. The exclusion criteria included an individual call rate < 95% and sex mismatch. Before imputation, variants were filtered by a call rate < 95%, HWE p value < 1e-4 (autosomal variants only), and minor allele frequency (MAF) < 1%. Prephasing was performed using Eagle v2.3 software (the number of conditioning haplotypes Eagle2 uses when phasing each sample was set to –Kpbwt = 20,000), and Beagle v.28Sep18.79339 with an effective population size ne = 20,000 was used for genotype imputation. A population-specific imputation reference of 2297 WGS samples was used [16].

The health data for EstBB participants were obtained from regular linking with the Estonian Health Insurance Fund (EHIF), the Estonian Cancer Registry (ECR), and the Causes of Death Registry, which are population-based and nationwide health/administration registries [17]. The EHIF is the core purchaser of health care services in Estonia, covering health care costs for insured people and managing services for uninsured citizens. The information on health status is stored as diagnostic codes based on the International Statistical Classification of Diseases and Related Health Problems 10th Revision (ICD-10) and codes relating to medical services and procedures with corresponding dates. As the EHIF reimburses health care providers on a fee-for-service basis, the database is considered to be relatively complete. As of December 2021, the EHIF contained information on 1 265 601 individuals or 94% of the Estonian population with insurance coverage [18]. The ECR is a population-based registry with nationwide coverage that has reliable cancer incidence data from 1968. It is compulsory for all physicians and pathologists working in Estonia to report cancer cases to the ECR. Additionally, the ECR uses multiple sources to ascertain cancer cases, including regular linkages with two cancer centres and trace-back of cases identified via death certificates. The completeness of case reporting is high, as evidenced by data quality indicators [19]. HPV data originated from a study in which an age-stratified (30–33, 57–60, 67–70) random sample of EstBB female gene donors was invited to a biobehavioural survey utilizing a self-administered survey on risk factors for cervical cancer and self-collected vaginal swabs for high-risk HPV detection (Additional file 1: Note and Additional file 2: Tables).

### Case and control definitions

In this study we employ a broad definition of the study outcomes that is informed by previous studies^11^. We have defined carcinoma in situ or invasive cervical cancer as cervical cancer, and CIN 2 or CIN3 as CIN. Women who had undergone hysterectomy were excluded from the analysis in both case and control cohorts. For the acquisition of cervical cancer cases we relied on data from both the ECR and the EHIF, as registry cases are reported with a two-year lag time. Both sources provided data based on diagnostic codes derived from the International Statistical Classification of Diseases and Related Health Problems 10th Revision (ICD-10).

### Cervical intraepithelial neoplasia (CIN)

Phenotypes CIN 2 and 3 were defined using data from EHIF with ICD-10 codes N87.1 (CIN2), N87.2 (CIN3) coinciding with procedure/Nomesco codes corresponding to histological evaluation or biopsy of the cervix on the same medical claim (Additional file 1). This dual-criteria approach was implemented to enhance the reliability of case classification and minimize any potential misclassification issues.

### Cervical cancer (CC)

Cervical cancer was defined using ECR and EHIF data with ICD-10 codes C53 and D06 and all their subcodes. Prevalent cervical cancer cases were defined as individual cancer patients who had received their diagnosis before joining EstBB. Incident cases of CIN and cancer were defined as individuals who were free of cervical cancer or CIN diagnosis at recruitment but received the corresponding diagnosis during the follow-up period.

Women who tested positive for any of the *high-risk HPV types* were considered to be infected.

### Control group women

Control group women were defined as women without cervical pathology who had a known Pap test status (normal cytological finding) (Additional file 1).

## Statistical analysis

Polygenic risk scoring methods.

### LDpred

We used the PRSs developed by Koel et al. [11], which were calculated using the LDPred software and a GWAS meta-analysis of UKBB, Kaiser Permanente, and FinnGen data (discovery sample). In brief, LDPred is a PRS software that adjusts GWAS summary statistics for the effects of linkage disequilibrium and produces different PRS profiles that differ in the expected proportion of causal SNPs and the adjusted weights given to individual SNPs. This set of PRSs included ten scores.

### Bayesian whole-genome regression (BayesRR-RC)

BayesRR-RC used Bayesian whole-genome regression approaches. We compared the PRSs based on the BayesRR-RC model [20] using case‒control data and the BayesW model [21] using time-to-event data. In contrast to LDpred, the SNP weights are recovered by analysing individual-level data. Hence, these models simultaneously estimate the effects of all variants, potentially giving an optimal predictor. The models were estimated for cervical cancer using only UK Biobank data of N = 248,798 European ancestry women (discovery sample), including 8680 cervical cancer cases and 2,174,071 SNPs [22]. All PRSs were standardized, and effect sizes corresponded to an increase by one standard deviation.

### Selecting the best-performing PRS from each score

The best PRSs were evaluated in a prevalence cervical cancer dataset comprising 691 prevalent cervical cancer case subjects and 13,820 control subjects. We tested the association between the PRS and the phenotype using age-adjusted logistic regression models. Based on the obtained odds ratios (ORs), we selected the best-performing PRSs from each test set and named them LDpred and BayesRR-RC.

### MetaPRS

To test the potential joint effect of LDpred and BayesRR-RC, we additionally combined them into a metaPRS [22], which was a weighted sum of the two scores. To construct the metaPRS, log odds ratios of PRSs from the logistic regression model in the prevalent analysis step were used as weights.

PRS association analysis with prevalent CIN and CC.

### Association with prevalent cervical intraepithelial neoplasia

Prevalent and incident cases represented slightly different aspects of the phenotype and were therefore analysed separately. Prevalent cases can be biased towards those with better survival, while incident cases represent the likelihood of getting the disease. Therefore, analysis of incident cases separately can allow a more thorough characterization of the predictive power of the PRS. We used the two best-performing PRSs identified in the first step of the analysis and tested their association with prevalent CIN cases in EstBB data. To test the association between the PRSs (LDpred and BayesRR-RC) and prevalent CIN, we used an age-adjusted logistic regression model comparing prevalent cases and controls (individuals without cervical pathology and with a known PAP test status). We then compared the *p* values and odds ratios (ORs) between the two scores.

### Association with incident cervical cancer and cervical intraepithelial neoplasia

Both PRSs (LDpred and BayesRR-RC) were evaluated in the analysis of incident CC and CIN cases and controls. This validation set was used to test the predictive ability of the PRSs. The PRSs were standardized and categorized into different groups of percentiles. We used Cox proportional hazard models to estimate the hazard ratios (HRs) corresponding to one standard deviation of the continuous PRS for the validation dataset. Harrell’s C-statistic was used to characterize the discriminative ability of each PRS estimated from the same Cox proportional hazard models. Cumulative incidence estimates accounting for competing events (mortality) were computed using the “cmprsk” R library. While comparing different PRS groups with each other, age was used as a timescale (using both age at entry and age at the end of follow-up/diagnosis) to properly account for left truncation in the data.

### Polygenic risk analysis and its association with hrHPV infection

A total of 1347 women responded to the questionnaire, 207 of whom were hrHPV positive (Additional file 2: Table S4). Medians and interquartile ranges are presented for the numerical variables. Percentages for each of the categories are presented within either hrHPV-positive or hrHPV-negative classes. We present odd ratios for the genetic and non-genetic predictors of hrHPV infection (Additional file 2: Table S2). The Akaike Information Criterion (AIC) was employed for the purpose of variable and best model selection. (Additional file 2: Table S3). Area under the ROC curve (AUC) for the logistic regression model that included (i) only 8 non-genetic predictor variables and (ii) also included cervical cancer PRS to predict HPV status were calculated. AUC was calculated by partitioning the full data set (n = 1339) to training (80%) and test data (20%) sets 100 times. The final AUC values were the means across 100 training-test partitions.

We used R 4.1.1 for analysis [23].

### Supplementary Information

**Additional file 1.** Supplementary Notes.

**Additional file 2.** Supplementary Tables 1–4.

## Data Availability

We do not have ethical approval to share individual-level genotype or phenotype data for the Estonian Biobank. Researchers interested in the Estonian Biobank can request access at https://www.geenivaramu.ee/en/access-biobank, and access to the UK Biobank can be requested at http://www.ukbiobank.ac.uk/resources/. The individual level data from Estonian Biobank are available under restricted access for containing sensitive information from healthcare registers, access can be obtained through the Estonian biobank upon submission of a research plan and signing a data transfer agreement. All data access to the Estonian Biobank must follow the informed consent regulations of the Estonian Committee on Bioethics and Human Research, which are clearly described in the Data Access section at https://genomics.ut.ee/en/content/estonian-biobank. A preliminary request for raw genetic and phenotype data must first be submitted via the email address releases@ut.ee. UKBB summary statistics can be accessed from http://www.decode.com/summarydata.
