# Supplementary material for: Polygenic risk scores for cervical HPV infection, neoplasia and cancer show potential for personalised screening: comparison of two methods
Source: Infect Agent Cancer. 2023 Dec 7;18:82. doi: 10.1186/s13027-023-00561-4 (PMC10702115; doi:10.1186/s13027-023-00561-4)
Supplement: Supplementary file 1 — Additional file 1. Supplementary Notes. [file 13027_2023_561_MOESM1_ESM.rtf]

Supplementary Notes

Definition of controls
As controls, we used women who had a Pap test (identified via Estonian Health Insurance Fund treatment bills with service codes 66807 and 66809) during the five previous years until the end of follow-up (either date of the last linking or time of death) and did not have any of the ICD-10 codes (D06, C53, N87.1, N87.2, O82.2 delivery by Caesarean hysterectomy, Z90.7 acquired absence of genital organs) or Nomesco procedure codes LCC Partial excision of uterus; LCC 00 Partial excision of uterus; LCC 01 Laparoscopic partial excision of uterus; LCC 05 Hysteroscopic excision of uterine wall; LCC 10 Supravaginal hysterectomy; LCC 11 Laparoscopic subtotal hysterectomy Laparoscopic supravaginal hysterectomy (LSH); LCC 20 Vaginal supravaginal hysterectomy; LCC 96 Other partial excision of uterus; LCC 97 Other laparoscopic partial excision of uterus; LCD Total excision of uterus Caesarean hysterectomy; LCD 00 Hysterectomy; LCD 01 Total laparoscopic hysterectomy; LCD 04 Laparoscopic hysterectomy; LCD 10 Vaginal hysterectomy; LCD 11 Laparoscopically assisted vaginal hysterectomy; LCD 30 Radical hysterectomy; LCD 31 Radical laparoscopic hysterectomy (RLH); LCD 40 Radical vaginal hysterectomy; LCD 96 Other hysterectomy; LCD 97 Other laparoscopic hysterectomy; LCE Exenteration of pelvis; LCE 00 Anterior exenteration of female pelvis; LCE 10 Posterior exenteration of female pelvis; LCE 20 Total exenteration of female pelvis; or LCE 96 Other exenteration of female pelvis.
Definition of CIN (service and procedure codes).
The procedure codes used included 6901 histology, routine; 6902 histology, moderately complicated differential diagnosis; 6903 histology, one extra staining; 6904 histology, express; 6905 histology, 5 or more extra stainings; 6906 immunohistology (1 monoclonal ab-s); 6907 histology (histochemical); 7004 biopsy; 7005 biopsy; LDA20 Biopsy of vaginal cervix; LDA96 Other biopsy of cervix uteri; LDB00 excision, cervical lesion; 71103 extirpation, cervical; LDC96 other excision of cervix uteri; LDW96 other operation on cervix uteri; and LDC10 partial excision of cervix uteri.
hrHPV infection
Vaginal samples collected during a cross-sectional study in 2021 using a self-sampling kit were analysed in the ISO 15 189 accredited diagnostic and national cervical cancer screening laboratory for HPV genotyping. Samples were analysed using the Luminex xMAP hrHPV assay, which detects 16 HPV types (16, 18, 31, 33, 35, 39, 45, 51, 52, 53, 56, 58, 59, 66, 68, 82) and is a clinically validated cervical cancer screening assay. The HPV genotypes were classified using the systems proposed by the International Agency for Research on Cancer (IARC), where twelve HPV types (16, 18, 31, 33, 35, 39, 45, 51, 52, 56, 58, 59) were classified as carcinogenic (high-risk) to humans, HPV type 68 as probably carcinogenic and HPV types 26, 53, 66, 67, 70, 73, 82 as possibly carcinogenic to humans. High-risk HPV (hrHPV) types included 16, 18, 31, 33, 35, 39, 45, 51, 52, 56, 58, 59, and 68.
