# Supplementary material for: Polygenic risk scores for cervical HPV infection, neoplasia and cancer show potential for personalised screening: comparison of two methods
Source: Infect Agent Cancer. 2023 Dec 7;18:82. doi: 10.1186/s13027-023-00561-4 (PMC10702115; doi:10.1186/s13027-023-00561-4)
Supplement: Supplementary file 2 — Additional file 2. Supplementary Tables 1–4. [file 13027_2023_561_MOESM2_ESM.rtf]

Supplementary Table 1. Evaluation of 12 PRSs calculated with two methods (best-performing scores marked in bold)
	OR	p-value	OR_L	OR_U	
LDpred.inf	1.173	5.34E-05	1.086	1.268	
LDpred_p1.0000e.00	1.225	2.87E-07	1.134	1.324	
LDpred_p1.0000e.01	1.239	6.31E-08	1.147	1.340	
LDpred_p1.0000e.02	1.360	2.24E-14	1.257	1.472	
LDpred_p1.0000e.03	1.278	8.43E-10	1.182	1.384	
LDpred_p1.0000e.04	1.164	9.15E-05	1.079	1.257	
LDpred_p3.0000e.01	1.228	2.02E-07	1.137	1.328	
LDpred_p3.0000e.02	1.276	8.13E-10	1.181	1.380	
LDpred_p3.0000e.03	1.443	1.59E-19	1.333	1.563	
LDpred_p3.0000e.04	1.106	1.07E-02	1.024	1.195	
BayesW	1.399	1.40E-16	1.292	1.516	
BayesRR-RC	1.445	1.54E-19	1.334	1.565	


Supplementary Table 2. Predictors of hrHPV status using LDpred or BayesRR-RC. Genetic risk has been standardized meaning that the OR characterizes the ratio of odds for subjects with 1 SD difference in their genetic risk score.
Variable (baseline)	OR (95% CI) Adjustment with BayesRR-RC	OR (95% CI) Adjustment with LDpred	
Genetic risk score	1.25 (1.08, 1.44)	1.26 (1.09, 1.46)	
Age group (30-33)			
57-60	0.75 (0.5, 1.12)	0.74 (0.5, 1.12)	
67-70	1.1 (0.65, 1.86)	1.1 (0.64, 1.89)	
Marital status (Married/Partnered)		
Single (widowed, divorced)	1.77 (1.29, 2.43)	1.81 (1.32, 2.5)	
Education (Tertiary education)1			
Primary education	2.02 (0.78, 5.24)	1.89 (0.72, 4.97)	
Secondary education	1.37 (1, 1.86)	1.38 (1.01, 1.88)	
Parity (0)			
1 or 2	0.63 (0.46, 0.86)	0.62 (0.46, 0.86)	
3 or more	0.52 (0.3, 0.9)	0.51 (0.29, 0.88)	
Hormonal contraceptives use (No)		
Yes	1.63 (1.06, 2.49)	1.63 (1.01, 2.63)	
Number of sex partners	1.04 (1.02, 1.06)	1.04 (1.02, 1.06)	
STI (No)			
Yes	1.57 (0.98, 2.51)	1.59 (1, 2.54)	
Awareness of PAP test interval (No)		
Yes	1.31 (0.93, 1.82)	1.31 (0.94, 1.83)	


1 Tertiary as third-level education after school


Supplementary Table 3. Akaike Information Criteria based model selection
Variable excluded from model	AIC (BayesRR-RC)	AIC (LDPred)	
(All variables included)	1266.368	1265.209	
Number of abortions	1265.043	1264.136	
Number of pregnancies	1263.114	1262.2	
Financial condition	1259.413	1258.414	
Contraceptives at first intercourse	1255.143	1253.815	
Age at first intercourse	1253.211	1251.962	
Last reproductive health exam	1251.075	1249.457	
Nationality	1250.786	1248.889	
Further exclusions did not result in AIC reduction	


Supplementary Table 4. Factors measured by the hrHPV infection status
		hrHPV-positive		hrHPV-negative		hrHPV-total	
	n		n		n		
Birth year, median, mean (IQR)	207	1988, 1975.5 
(1961-1989)	1140	1963, 1972.3
(1960-1989)	1347	1963, 1972.8 (1960-1989)	
Age of first intercourse, median, mean (IQR)	202	18, 18.3
(16-20)	1062	18, 18.7 
(17-20)	1264	18, 18.7 
(17-20)	
Number of pregnancies, median, mean (IQR)	140	3, 3.1 
(2-4)	888	3, 3.1 
(2-4)	1028	3, 3.1 
(2-4)	
Age at pregnancy, median, mean (IQR)	134	22, 23.1 (20-27)	861	23, 23 (20-26)	995	22, 23 (20-26)	
Number of sex partners, median, mean (IQR)	193	6, 8 (4-10)	1038	4, 5.5 (2-6)	1231	4, 5.9 (2-7)	
Number of abortions, median, mean (IQR)	207	0, 0.7 (0-1)	1140	0, 0.9 (0-1.25)	1347	0, 0.9 (0-1)	
Marital status, n (%)	207		1138		1345		
Married/Partnered		127 (61%)		811 (71%)		938 (70%)	
Single (widowed, divorced)		80 (39%)		327 (29%)		407 (30%)	
Education, n (%)1	205		1133		1338		
Tertiary education		116 (57%)		670 (59%)		786 (59%)	
Secondary education		82 (40%)		446 (39%)		528 (39%)	
Primary education		7 (3%)		17 (2%)		24 (2%)	
Contraceptives at first intercourse, n (%)	199		1055		1254		
Condom		98 (49%)		426 (40%)		524 (42%)	
Hormonal/Intrauterine/Emergency pill		11 (6%)		50 (5%)		61 (5%)	
Nothing		69 (35%)		459 (44%)		528 (42%)	
Other		21 (11%)		120 (11%)		141 (11%)	
STI, n (%)	199		1062		1261		
No		173 (87%)		957 (90%)		1130 (90%)	
Yes		26 (13%)		105 (10%)		131 (10%)	
Last reproductive health exam, n (%)	203		1125		1328		
< 1 year ago		110 (54%)		486 (43%)		596 (45%)	
1-2 years ago		36 (18%)		261 (23%)		297 (22%)	
2-3 years ago		20 (10%)		128 (11%)		148 (11%)	
3+ years ago		27 (13%)		173 (15%)		200 (15%)	
Never or did not know		10 (5%)		77 (7%)		87 (7%)	
Has used hormonal contraceptives, n (%)	196		1045		1241		
No		66 (34%)		477 (46%)		543 (44%)	
Yes		130 (66%)		568 (54%)		698 (56%)	
PAP test interval awareness, n (%)	206		1120		1326		
No		59 (29%)		350 (31%)		409 (31%)	
Yes		147 (71%)		770 (69%)		917 (69%)	
Financial condition, n (%)	207		1140		1347		
Average		123 (59%)		672 (59%)		795 (59%)	
Bad		27 (13%)		148 (13%)		175 (13%)	
Good		57 (28%)		320 (28%)		377 (28%)	
Nationality, n (%)	207		1140		1347		
Estonian		168 (81%)		936 (82%)		1104 (82%)	
Other		39 (19%)		204 (18%)		243 (18%)	
Parity, n (%)	207		1140		1347		
0		100 (48%)		376 (33%)		476 (35%)	
1-2		91 (44%)		624 (55%)		715 (53%)	
3		16 (8%)		140 (12%)		156 (12%)	
Age group, n (%)	207		1140		1347		
30-33		120 (58%)		523 (46%)		643 (48%)	
57-60		48 (23%)		422 (37%)		470 (35%)	
67-70		39 (19%)		195 (17%)		234 (17%)	
